# Clinical and Pathologic Features of Out-of-Hospital Cardiac Arrest in Pregnancy: Insights From a Statewide Registry

**DOI:** 10.1016/j.jacadv.2022.100049

**Published:** 2022-08-22

**Authors:** Elizabeth D. Paratz, Stephanie Rowe, Alexander van Heusden, Karen Smith, Andreas Pflaumer, Christopher Semsarian, Sarah Parsons, Dion Stub, Dominica Zentner, Andre La Gerche

Cardiovascular disorders are the leading cause of indirect maternal mortality worldwide, with sudden cardiac arrest estimated to occur in approximately 1 in 12 to 30,000 pregnancies.^1^ Only 2 studies to date have evaluated rates and outcomes of out-of-hospital cardiac arrest (OHCA) in pregnancy specifically, with a cumulative total of 22 pregnant OHCA patients.^2^^,^^3^ There is a need for more data on OHCA in pregnancy; however, systematic case identification is challenging as pregnancy status is not collected in standardized Utstein templates in OHCA registries. This study identified rates, causes, and outcomes of OHCA in pregnancy or <3 months postpartum over the time period April 2019 to April 2021 in the state of Victoria, Australia.

The EndUCD (End Unexplained Cardiac Death) registry is a statewide prospective multisource surveillance cardiac arrest registry identifying all OHCA cases in the state of Victoria, Australia, (population 6.7 million). All OHCA cases 1 to 50 years old attended by Ambulance Victoria are identified with subsequent case adjudication against hospital and forensic data. The registry holds ethical approval via the Alfred Hospital Human Research Ethics Committee (approval number 567/18).

Age- and sex-specific information regarding the number of residents in Victoria, Australia, throughout the study period was obtained from governmental data sets. Consistent with nationally-defined criteria, females aged 15 to 49 years were considered “of child-bearing age”.^3^ Data regarding the number of females who either gave birth or were within 3 months postpartum during the defined study period of April 2019 to April 2021 were provided by the Data and Analytics Department of the Victorian Agency for Health Information.

During the study period, 154,914 females were pregnant in Victoria. Eight females experienced an OHCA either while being pregnant or immediately postpartum (incidence rate: 5.2 per 100,000 pregnancies). Of 1,627,056 females of child-bearing age (15-49 years old),^4^ 376 females suffered a cardiac arrest (incidence rate: 23.1 per 100,000 females of child-bearing age) (Figure 1).

**Figure 1:**
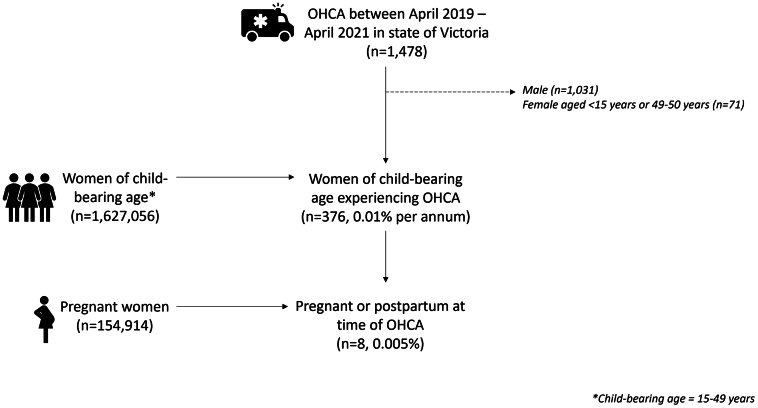
Diagram Indicating Number of OHCAs and Number of Pregnant Females With OHCA in the Context of Overall Pregnancies OHCA = out-of-hospital cardiac arrest.

Among patients who experienced OHCA, the average patient age was 29.9 years (range: 19.8-39.8 years). One patient was 8 weeks postpartum, while the other 7 patients were all pregnant (gestational range: 8-22 weeks). Two females were known to have obesity before OHCA, but no other cardiovascular risk factors had been identified. One patient had a history of amphetamine and alcohol abuse. No patients had ever attended a cardiologist or a specialized cardiac pregnancy clinic as part of antenatal evaluation.

Five of the 8 OHCAs were witnessed; however, only 3 patients (60%) received bystander cardiopulmonary resuscitation. A shockable cardiac rhythm (ventricular fibrillation) was identified in 2 patients who both (100%) received defibrillation. The remaining patients had nonshockable rhythms with 2 patients having pulseless electrical activity and 4 having asystole on presentation. Four patients (50%) were transported to hospital, with the remainder declared deceased at the scene. Only 1 patient survived to hospital discharge (12.5%). As no pregnancies were of viable age, no perimortem caesarean sections were performed.

Five of the 7 deceased patients (71.4%) proceeded to autopsy examination. Postmortem toxicology was positive for cannabis in 1 patient (who died from nonischemic cardiomyopathy) and negative for illicit drugs in all remaining patients. Five of the 8 OHCAs (62.5%) were determined to be cardiac in etiology; unascertained (n = 2), hypertrophic cardiomyopathy (n = 1), nonischemic cardiomyopathy (n = 1), and ischemic heart disease (n = 1). Three OHCAs had a noncardiac cause, with 2 cases of intracerebral hemorrhage and 1 case of splenic artery aneurysm. No OHCAs were directly attributable to the pregnancy.

To summarize, this study identified that OHCA occurs in approximately 1 in 20,000 pregnancies, with high maternal mortality. OHCA is commonly due to cardiac causes but with low rates of prearrest diagnosis.

Our paper is only the third to report on outcomes of OHCA in pregnancy and the first to utilize comprehensive hospital and forensic adjudication for all cases to ensure wide-ranging case capture.

International coding criteria recommend defining episodes of maternal mortality or cardiac arrest as being “direct” (resulting from complications of the pregnant state), “indirect” (those not due to a direct obstetric cause but potentially aggravated by the physiological state of pregnancy), or “coincidental” (deaths from unrelated causes). In our cohort, all OHCAs were due to an indirect cause, with 62.5% of OHCAs due to a cardiac cause. Ambulance-based OHCA registries do not routinely identify pregnancy status as an element of the Utstein template. Given the limited data to date on OHCA in pregnancy and its apparent distinct clinical profile, the routine addition of pregnancy status to prehospital data collection may be worthy of consideration.

It has been estimated that up to 68% of cardiac pregnancy deaths are avoidable,^5^ and therefore, early recognition of patients with cardiovascular disease is key. Unfortunately, the paradox of young OHCA is that the majority of events occur in those classed as low risk and those with unrecognized underlying conditions. In our cohort, this pattern is strongly represented. Although 2 patients were obese, this would not typically precipitate a referral to a pregnancy heart team. Indeed, no patients in our study had seen a cardiologist or pregnancy heart team or had any formal cardiac diagnosis in life. Two OHCAs remained unascertained after comprehensive postarrest investigations. Identifying patients who are ostensibly at low risk but will suffer an OHCA in pregnancy remains an ongoing challenge.

The major limitation in this study is the small number of pregnant females experiencing OHCAs; however, this is commensurate with reported numbers and rates of pregnancy OHCA in other studies. The COVID-19 pandemic during this timeframe may have impacted on prenatal care delivery and cardiac screening.

In conclusion, maternal OHCA affects approximately 1 in 20,000 pregnancies, with high maternal mortality rates. Approximately two-thirds of maternal OHCA cases have an underlying cardiac cause, but with low rates of cardiac diagnosis before arrest. Identifying pregnancy status routinely in OHCA patients would support global data collection.

## References

[1] Beckett V.A., Knight M., Sharpe P. (2017). The CAPS study: incidence, management and outcomes of cardiac arrest in pregnancy in the UK: a prospective, descriptive study. BJOG.

[2] Maurin O., Lemoine S., Jost D. (2019). Maternal out-of-hospital cardiac arrest: a retrospective observational study. Resuscitation.

[3] Lipowicz A.A., Cheskes S., Gray S.H. (2018). Incidence, outcomes and guideline compliance of out-of-hospital maternal cardiac arrest resuscitations: a population-based cohort study. Resuscitation.

[4] Australian Bureau of Statistics (2021). https://www.abs.gov.au/statistics/people/population/national-state-and-territory-population/mar-2021.

[5] Ouyang P., Sharma G. (2020). The potential for pregnancy heart teams to reduce maternal mortality in women with cardiovascular disease. J Am Coll Cardiol.

